# Monkeypox in Healthcare Settings: A Narrative Review of Transmission, Exposure, and Protection Among Healthcare Workers

**DOI:** 10.7759/cureus.101286

**Published:** 2026-01-11

**Authors:** Haseeb Mehmood Qadri, Saad Abdullah Dar, Amara Gondal, Hafiz Sohail Ahmad, Maira Jabbar Chaudhry, Salamat Ali, Shiza Rashid, Saqiba Khan, Syeda Gul e Zehra Zaidi, Nusrat Fatima

**Affiliations:** 1 General Surgery, Lahore General Hospital, Lahore, PAK; 2 Neurosurgery, Punjab Institute of Neurosciences, Lahore, PAK; 3 Internal Medicine, Aziz Bhatti Shaheed Teaching Hospital, Gujrat, PAK; 4 Pediatric Surgery, University of Child Health Sciences, Children Hospital Lahore, Lahore, PAK; 5 Pathology, Azra Naheed Medical College, Lahore, PAK; 6 General Surgery, Aziz Bhatti Shaheed Teaching Hospital, Gujrat, PAK; 7 Internal Medicine, Nawaz Sharif Medical College, Gujrat, PAK; 8 Obstetrics and Gynecology, Gulab Devi Teaching Hospital, Lahore, PAK; 9 General Surgery, Bait Ul Shifa Clinic, Lahore, PAK; 10 Respiratory Medicine, Portsmouth Hospitals University NHS Trust, Portsmouth, GBR

**Keywords:** hand hygiene, health personnel, humans, monkeypox, needlestick injuries, vaccination

## Abstract

Monkeypox, caused by an Orthopoxvirus, poses a significant occupational risk to healthcare workers due to their frequent contact with infected patients and contaminated materials. This risk is increased by potential lapses in infection control protocols in hospitals, such as a lack of personal protective equipment (PPE), improper hand hygiene, and other issues, making hospitals a key site for occupational transmission of monkeypox. The objective of the study was to evaluate monkeypox transmission among healthcare workers and identify critical preventive strategies. A comprehensive literature search was conducted using PubMed, focusing on case reports and series published between 2000 and 2024. Eight studies comprising seven case reports and one case series, documenting a total of nine patients, were selected based on predefined inclusion criteria. Data regarding transmission routes, risk factors, and preventive measures were extracted and analyzed.

The primary mode of monkeypox transmission among healthcare workers was percutaneous exposure, notably from needlestick injuries during the care of patients with active lesions. Fomite exposure accounted for 22.22% (2) of cases, highlighting the risk posed by contaminated surfaces and medical equipment. Respiratory transmission was suspected in some cases, though conclusive evidence remains limited. Key risk factors included inadequate use of PPE and breaches in infection control protocols. Post-exposure vaccination was administered to 33.33% (3) of patients, and post-exposure medication was given to 66.66% (6) of patients.

Although respiratory transmission is not evident, asymptomatic or mildly symptomatic individuals may contribute to disease transmission. This highlights the need for improved surveillance and enhanced infection control protocols, including PPE utilization, hand hygiene, and avoidance of needlestick injuries. Post-exposure vaccination and antiviral treatment of exposed individuals effectively curb the spread of monkeypox.

## Introduction and background

The arrival of the monkeypox virus has raised worldwide interest. It was first isolated from laboratory monkeys in Denmark in 1958 [1]. The monkeypox virus is a double-stranded DNA virus within the Poxviridae family, subcategorized as Orthopoxvirus. It's sustained via numerous animal reservoirs, including rodents, monkeys, squirrels, Gambian pouched rats, dormice, and non-human primates [1-3]. Its mode of transmission to humans is via bites, scratches, near-contact, ingestion of undercooked meat from infected animals, inhalation of respiratory droplets, direct contact with infectious fluids, and contaminated surfaces [1,2]. Vertical transmission from mother to fetus has also been reported [1,4].

The monkeypox virus presents clinically with a rash that evolves through macules, papules, vesicles, and pustules, preceded by prodromal signs and symptoms, including fever, lymphadenopathy, and flu-like symptoms [1,2]. Patients are considered to be infectious from the onset of prodrome or rash until the lesions scab over. Diagnosis is established by polymerase chain reaction detection of viral DNA, and most patients recover within two to four weeks with symptomatic treatment. It is usually recommended to immunize high-risk groups and individuals with smallpox vaccines (JYNNEOS™ and ACAM2000®) to provide protection against this virus and improve clinical outcomes [1,2]. The vaccinia immune globulin and antivirals are recommended for severely affected and immunocompromised patients.

Genomic research categorizes the monkeypox virus into two clades: African/Congo Basin and West African. The West African clade, associated with most outbreaks outside Africa, generally causes mild disease [3]. Conversely, the African/Congo Basin clade is more virulent and has a high fatality rate [1]. Human-to-human transmission occurs through large respiratory droplets, direct skin or mucosal contact, and contaminated objects. Although sexual transmission is not fully established, close physical contact during sexual activity can enable the spread [1].

Given that, in 2022, monkeypox became an international challenge, and in July, the World Health Organization (WHO) declared it a public health emergency of international concern [4]. Our review focuses on the significant health risk this virus poses to different occupational workers. Our main goal was to understand its transmission among different occupational setups, especially among healthcare workers, including doctors, nurses, and laboratory personnel. Moreover, an unsafe working environment, an inadequate supply of personal protective equipment (PPE), high staff-to-patient ratios, and direct contact with infected patients, body fluids, and contaminated materials may increase the risk [5,6]. Additionally, veterinarians, animal handlers, public health workers, and employees in the travel and hospitality industries are also at high risk. We need to address these risks, discuss effective controls and cutting-edge preventive measures, establish useful policies, and implement adequate education on its spread.

There is a significant literature gap on the monkeypox virus due to its limited research priority as compared to other well-known viruses like HIV, Zika, and Ebola; its regional confinement to Central and West Africa; and its being a rare and mild disease. Moreover, underreporting and limited surveillance, combined with resource constraints, contributed to this gap. This article addresses that gap by studying the occupational spread of this virus and providing clinical recommendations to control its further spread.

## Review

Methodology

This narrative review aimed to characterize the modes of transmission, clinical manifestations, and geographic distribution of the monkeypox virus among healthcare workers.

Search Strategy

A thorough literature search was conducted using the electronic database PubMed. The search was conducted using Boolean operators (“AND” and “OR”) and incorporated key terms and phrases including “monkeypox virus”, “monkeypox transmission”, “occupational infection”, “needlestick injury”, “occupational exposure”, and “outbreak”.

Inclusion and Exclusion Criteria

All case reports and case series published in English between 2000 and 2024 that contained data on the occupational spread of monkeypox virus among healthcare professionals and paramedical staff were retrieved. Letters to editors, editorials, non-English language articles, articles containing non-human sources, and clinical images were excluded.

Data Extraction and Synthesis

Eight studies were selected to work on the set objective. Key information from the selected studies, including study design, population and setting, risk factors, transmission routes, and preventive measures, was extracted by three reviewers (A.G., S.R., S.K.) who read all the articles independently. The findings were then synthesized to highlight common themes, trends, and knowledge gaps. The data were then verified for completeness and accuracy and harvested into a Microsoft Office 365 Word (Microsoft Corp., Redmond, WA, USA)-generated pro forma. Descriptive analysis was employed to compile the results. The details of all included studies are summarized in Table 1.

**Table 1:** Details of included studies

Study by	Study title	Year of publication	Country of publication	Study design
Choi et al. [7]	Occupational transmission of monkeypox virus to healthcare workers	2022	South Korea	Case report
Alarcon et al. [8]	Occupational monkeypox virus transmission to healthcare worker	2022	USA	Case report
Caldas et al. [9]	Monkeypox after occupational needlestick injury from pustule	2022	Portugal	Case report
Mendoza et al. [10]	Monkeypox virus infection resulting from occupational needlestick	2022	USA	Case report
Salvato et al. [11]	Possible occupational infection of healthcare workers with monkeypox virus	2022	Brazil	Case series
Carvalho et al. [12]	Monkeypox virus transmission to healthcare worker through needlestick injury	2022	Brazil	Case report
Safir et al. [13]	Nosocomial transmission of monkeypox virus to healthcare workers	2022	Israel	Case report
Migaud et al. [14]	Occupational transmission of mpox	2023	Germany	Case report

Results

This study included eight studies, one case series, and seven case reports, documenting a total of nine patients, two in the case series and one in each case report, as given in Table 2.

**Table 2:** Summary of included studies, showing distribution by study design, with the number of studies (n) and the total number of patients (N)

Study type	Number of studies, n	Number of patients (N)
Case reports	7	7
Case series	1	2
Total	8	9

Among the documented population, 77.78% (7) were adults, and there were no pediatric cases; the remaining 22.22% (2) was not specified, as elaborated in Table 3.

**Table 3:** Distribution of patients by age group along with their percentage (%)

Age group	Number of cases, n (N = 9)	Percentage occurrence (n/N)
Adults	7	77.78%
Pediatric	0	0%
Not given	2	22.22%

Regarding gender distribution, 33.33% (3) were male, and 44.44% (4) were female. For 22.22% (2), neither the age group nor the gender was specified, as shown in Table 4. The mean age and standard deviation of males and females were 32.33 ± 3.40 years and 30.25 ± 7.29 years, respectively (Table 5).

**Table 4:** Gender distribution of included patients along with their percentage (%)

Gender	Number of cases, n (N = 9)	Percentage occurrence (n/N)
Male	3	33.33%
Female	4	44.44%
Not specified	2	22.22%

**Table 5:** Mean age (years) and standard deviation of the included patients

Age group	Mean age (years)	Standard deviation
Males	32.33	3.40
Females	30.25	7.29

Globally, 33.33% (3) of the cases were reported from Brazil, another 22.22% (2) from the USA, and 11.11% (1) from Portugal, as represented in Table 6.

**Table 6:** Geographic distribution of included studies (top three countries)

Country	Number of studies, n (N= 9)	Percentage Occurrence (n/N)
Brazil	3	33.33%
USA	2	22.22%
Portugal	1	11.11%

As mentioned in Tables 7-8, in 22.22% (2) of the cases, the presenting complaint was vesicle formation, 22.22% (2) presented with papules, and another 11.11% (1) had raised lesions. Blisters, macules, nodules, and whitish spots were each observed in 1% of cases (7). Additionally, 11.11% had unspecified skin lesions, and 55.55% (9) presented with systemic symptoms, including malaise, headache, cough, and myalgias; fever and lymphadenopathy were also reported.

**Table 7:** Major presenting signs of patients along with their percentage occurrence (%)

Signs at presentation	Number of cases, n (N = 9)	Percentage occurrence (n/N)
Vesicle on the infected area or site of lesion	2	22.22%
Papule on the skin	2	22.22%
Raised skin lesion with or without progression to blister	1	11.11%
Blister on the nose	1	11.11%
Macula with central umblication	1	11.11%
Nodule at the site of injury	1	11.11%
Whitish spot on the index finger	1	11.11%
Unspecified skin lesion at the site of needlestick injury	1	11.11%
No specific signs (instead presented with systemic signs)	1	11.11%

**Table 8:** Presenting symptoms of patients along with their percentage occurrence (%)

Systemic signs and symptoms	Number of cases, n (N = 9)	Percentage occurrence (n/N)
Malaise and/or myalgia with/without fatigue, cough, and headache	5	55.56%
Fever	4	44.44%
Lymphadenopathy	3	33.33%
Lymphangitis	1	11.11%
Nil/not given	2	22.22%

Table 9 shows that percutaneous/nosocomial transmission was the most common mode of transmission. Fomite exposures were also identified as causes of the disease.

**Table 9:** Mode of transmission/source of infection of the disease

Mode of transmission/source of infection	Number of cases, n (N = 9)	Percentage occurrence (n/N)
Percutaneous transmission/nosocomial	7	77.77%
Fomite exposure	2	22.22%
Not mentioned	2	22.22%

The majority (55.55%) of cases involved a left-sided index finger lesion, with one patient presenting with a necrotic index finger as a complication of the disease, as given in Tables 10-11.

**Table 10:** Complications of the disease process and their percentage occurrence (%)

Complications	Number of cases, n (N = 9)	Percentage occurrence (n/N)
Necrotic index finger	1	11.11%
Nil	8	88.89%

**Table 11:** Lesion distribution by side and their percentage occurrence (%)

Side of lesion	Number of cases, n (N = 9)	Percentage occurrence (n/N)
Left-sided	5	55.55%
Right sided	1	11.11%
Bilateral	1	11.11%
Not mentioned	2	22.22%

Post-exposure vaccination was administered to 33.33% (3) of patients, and post-exposure medication was given to 66.66% (6) of patients (Tables 12-13). The mean inoculation period for all the patients in our review was 4.81 ± 2.44 days.

**Table 12:** Vaccine given to patients after exposure to monkeypox and its percentage (%)

Post-exposure vaccine	Number of cases, n (N = 9)	Percentage occurrence (n/N)
Yes	3	33.33%
No	6	66.66%

**Table 13:** Medicine given to patients after exposure to monkeypox and its percentage (%)

Post-exposure medicine	Number of cases, n (N = 9)	Percentage occurrence (n/N)
Yes	4	44.44%
No	5	55.55%

Discussion

Monkeypox primarily spreads through direct contact with infected individuals’ bodily secretions, such as respiratory droplets, bodily fluids, or contaminated materials. Healthcare workers are the population that comes into direct contact with infected individuals or contaminated materials, such as body fluids and fluid from lesions [6,15,16]. Transmission through sexual contact has also been established [6]. Our review identified percutaneous transmission as the predominant route, comprising 77.77% (7) of cases, reflecting the common occurrence of needlestick injuries in healthcare environments. These injuries commonly occur during procedures such as withdrawing samples and caring for patients with open lesions, where bodily fluids containing the virus can easily come into contact with the skin of healthcare personnel [9]. In our study, fomite exposure accounted for 22.22% (2) of cases, suggesting that contaminated materials and surfaces also play a role in transmission [11]. This finding underscores the importance of rigorous disinfection and cleaning protocols in healthcare settings. Materials such as clothes and equipment that come into contact with the patient’s skin lesions or vesicular fluids can serve as a source of transmission if not properly sanitized. The risk of monkeypox transmission via contaminated items is considerable when adherence to infection control protocols is poor [8,16]. A study found that healthcare workers were exposed to various occupational hazards, including blood-borne pathogens, chemicals, biologic hazards, and waste [17]. However, adherence to safety protocols among healthcare workers was poor, with reported rates of around 65.8% in inpatient settings, 67.5% in outpatient settings, 77.6% in surgery and anesthesia, 80.0% in nutrition services, and 68.9% in hospital support services departments [17].

Furthermore, transmission through respiratory droplets also plays some role, especially in places where close contact is common, and aerosols generated during procedures can be a potential source of spread. While the monkeypox virus is far less contagious than influenza or COVID-19, healthcare workers are still at risk when having close contact with infected patients. However, there is no conclusive evidence indicating this route of transmission, which invites further research [18].

Now, monkeypox cases among healthcare workers in nonendemic areas provide insights into the extensive epidemiological trends of its spread. The studies included in our review were conducted in Brazil and the USA (33.33%, 3), Portugal (11.11%, 1), and the USA (22.22%, 2) [12]. The higher number of cases reported in these regions is due to several reasons. The first monkeypox case was detected in the USA in 2003 [16]. In the USA, recently developed diagnostic tools, efficient surveillance systems, and advanced reporting mechanisms likely contribute to the higher case detection rate. The spread in the USA in the common population was seen in people who were in close contact with infected individuals, like sex workers and homosexual males [5,16]. Whereas in Brazil, the monkeypox spread is exacerbated by challenges such as limited public awareness, insufficient vaccination coverage, and insignificant infection control practices. The differences in healthcare infrastructure, public health awareness and responses, and access to preventive measures across the above countries suggest that monkeypox transmission among healthcare workers is strongly linked to the implementation and availability of healthcare resources [1].

The gender distribution was not significant, with 44.44% (4) female and 33.33% (3) male, and 22.22% (2) cases did not specify a gender. Age-wise, infected individuals were on average 31-32 years old, indicating that the virus primarily affects those in the peak of their professional careers [9].

Monkeypox presents with a variety of symptoms, many of which are characteristic of the disease’s progression. Vesicles on the involved skin site were the most common presentation, occurring in 22.22% (2) of cases, followed by papules, accounting for 22.22% (2) of cases [6]. These findings align with the typical progression of monkeypox, in which skin lesions are among the primary signs of infection. Other symptoms included blisters and nodules at the site of exposure, particularly at sites of needlestick injuries, further emphasizing the risk of percutaneous transmission [8,9,11]. Other systemic symptoms were also present, like malaise, myalgia, and fever, reported in 55.56% (5) and 44.44% (4) of cases, respectively [16]. These non-specific systemic symptoms are mostly the first indicators of infection, after which more visible lesions appear.

Regarding signs, lymphadenopathy was present in 33.33% (3) of the cases and is considered a significant finding of monkeypox infection. This illustrates the potential severity of monkeypox when left untreated. Although 88.89% of cases did not report severe complications, this indicates that most infections resolve without lasting damage [8,9].

The mean inoculation period was 4.81 ± 2.44 days, consistent with previous studies on monkeypox [6]. This relatively short incubation period underlines the importance of rapid identification and isolation of cases in healthcare settings to prevent further spread [9,11,12].

Infection control is essential for preventing the spread of monkeypox among healthcare workers. According to the guidelines, the use of PPE, including gloves, gowns, masks, and face shields, is essential for reducing the risk of transmission. Ensuring that healthcare workers have access to appropriate PPE and are trained in its proper use is crucial for reducing occupational exposure to monkeypox. Regular cleaning of high-contact surfaces and proper disposal of contaminated materials are key practices in preventing its transmission. Additionally, healthcare facilities should implement regular training programs to educate workers on the hazards of monkeypox [3].

This review found that only 33.33% (3) of cases received post-exposure vaccination, and 55.55% (6) received post-exposure medication, revealing significant gaps in preventive strategies [5]. These gaps are particularly noticeable in resource-shortage settings, where access to PPE, vaccines, and antiviral medications may be limited [6,8,9].

The limited availability of post-exposure vaccination shows the need for broader distribution of vaccines, particularly in areas with a high risk of monkeypox transmission, such as among HIV-infected individuals [4]. Although monkeypox is not as widespread as other infectious diseases, such as influenza or COVID-19, vaccination can play a critical role in reducing the incidence of healthcare-associated infections, especially among workers regularly exposed to infected patients [6].

Postexposure prophylaxis against HIV and antiviral treatment for influenza provide some insight into the prophylaxis of monkeypox. Ensuring that healthcare workers have timely access to these interventions could slow disease progression within healthcare settings [2,3]. Another example is TB, for which effective prevention and control protocols help establish a model for the prevention of monkeypox transmission in healthcare workers. Furthermore, integrating surveillance systems, as seen in the management of TB and HIV, could improve early detection and containment of monkeypox outbreaks in healthcare facilities [3].

**Figure 1:**
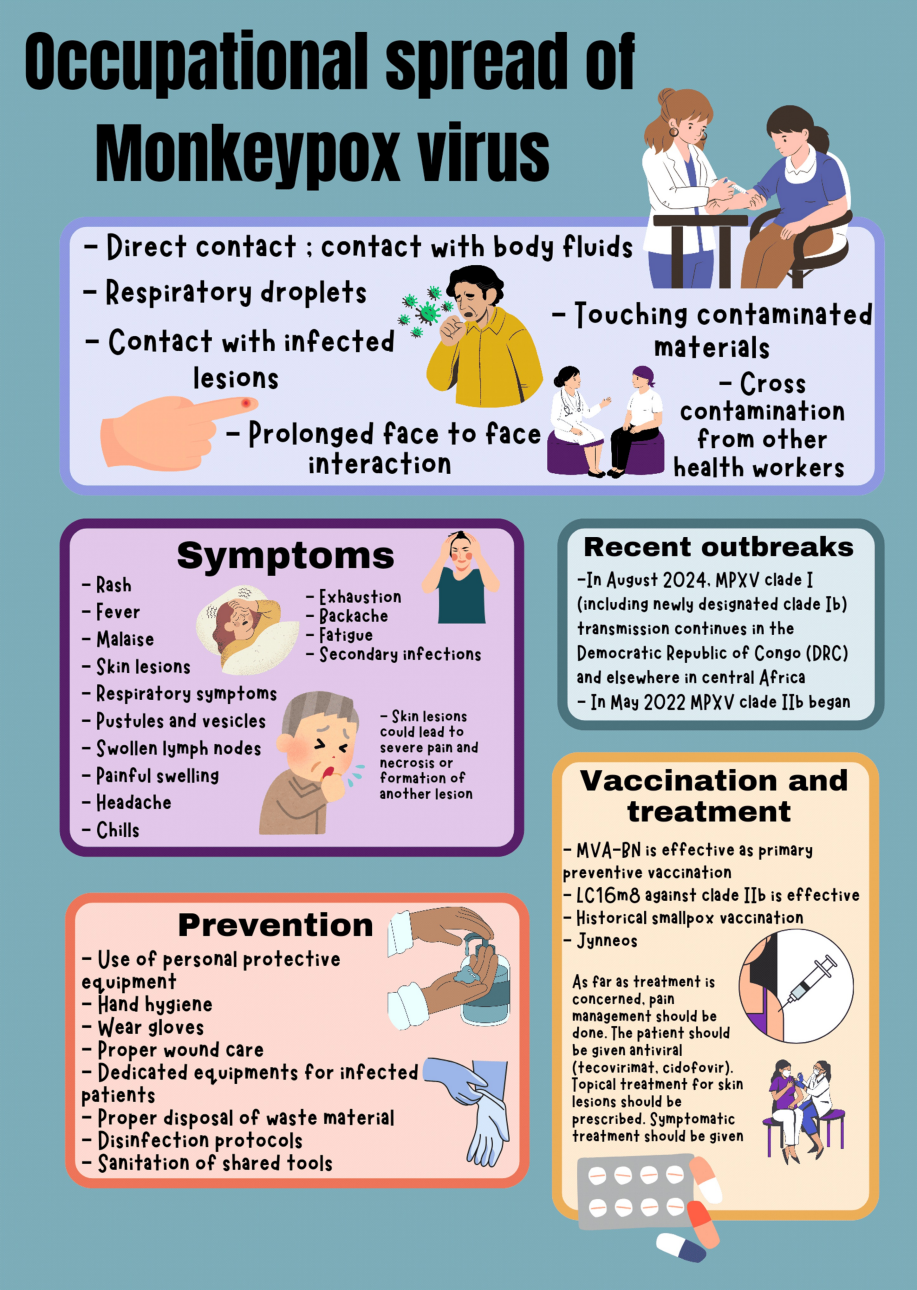
Author-proposed illustration of the occupational spread of monkeypox virus and prevention

As the WHO declared monkeypox a public health emergency, extensive guidelines should be established to prevent and control monkeypox transmission. PPE should be made available, especially in resource-limited environments where access to standard equipment may be compromised. The success of vaccination programs for diseases like hepatitis B and influenza highlights the importance of immunization in protecting healthcare workers [4]. Although monkeypox vaccination is not yet widely implemented, expanding access, particularly in high-risk areas, could significantly reduce the incidence of occupational transmission. Further research is needed to clarify the role of respiratory droplets in monkeypox transmission and to determine the optimal infection control measures to prevent this mode of transmission. Investigating the effectiveness of different disinfection protocols for fomite control could help reduce indirect transmission, particularly in settings where thorough cleaning is challenging [9,11,16]. A proposed illustration of the occupational spread of the monkeypox virus and prevention is given in Figure 1.

Limitations

This narrative review has several limitations. First, we included only seven case reports and one case series. Our study focused exclusively on adults due to limited data on children. Most of the case studies are from Brazil, the USA, and Portugal, indicating a lack of uniformity in data across regions. Additionally, since the primary route of monkeypox transmission is direct contact, often sexual, the history of coinfection with sexually transmitted diseases and its relation to the monkeypox virus has not yet been thoroughly studied. The connection between HIV and monkeypox is also underexplored, leaving the exact burden of monkeypox among patients with HIV unknown.

Clinical Recommendations

This narrative review article states that healthcare workers and individuals in certain occupations are at higher risk of contracting monkeypox. Several critical measures can be taken to reduce the spread of this virus. First, perfecting hand hygiene is essential, as most infections spread via hands through direct contact with an infected person or the environment. Second, the use of appropriate PPE and adequate disinfection and cleaning of the contaminated surfaces is also essential.

Although there is currently no specific monkeypox virus vaccine, smallpox vaccines (JYNNEOS™ and ACAM2000®) may provide some protection against monkeypox. Healthcare workers who are ill with respiratory tract infections and rashes should avoid patient duties. Furthermore, avoiding needlestick injuries, pre-employment vaccinations, and screening for common diseases (such as HIV, hepatitis B, and hepatitis C) is essential to prevent the spread. Finally, educational assessment and regular feedback are effective strategies for promoting preventive care.

## Conclusions

This review highlights the occupational hazard of monkeypox transmission among healthcare workers through needlestick injuries and fomite exposure, though respiratory transmission may also occur. Post-exposure prophylaxis and vaccination are significantly effective in halting its transmission. Addressing the known risk factors, particularly percutaneous and fomite transmission, through improved PPE use, effective training, and access to post-exposure vaccination can help prevent monkeypox transmission in healthcare settings. By implementing targeted interventions, healthcare facilities can better protect against monkeypox transmission and reduce its overall burden.
